# Supplementary material for: Co-Supplementation of Diet with Saccharomyces cerevisiae and Thymol: Effects on Growth Performance, Antioxidant and Immunological Responses of Rainbow Trout, Oncorhynchus mykiss
Source: Animals (Basel). 2025 Jan 22;15(3):302. doi: 10.3390/ani15030302 (PMC11815758; doi:10.3390/ani15030302)
Supplement: Supplementary file 1 [file animals-15-00302-s001.zip › animals-3437804-supplementary.pdf]

Gut RNA extraction and cDNA synthesis

The fish gut samples were used for RNA extraction, using a commercial kit (Denazist Co., Tehran, Iran). After treating with DNase I (Thermo Fisher Scientific, Waltham, MA, USA), the quality of RNA was approved by Nanodrop (Thermo Scientific, Nanodrop, 2000C, USA) and agarose gel (1%). Then, cDNA was synthetized using a commercial kit supplied by SMOBIO Technology Co. (Hsinchu City 30075, Taiwan) and used for rt-PCR reactions.
